# Optimised path planning using Enhanced Firefly Algorithm for a mobile robot

**DOI:** 10.1371/journal.pone.0308264

**Published:** 2024-08-12

**Authors:** Mohd Nadhir Ab Wahab, Amril Nazir, Ashraf Khalil, Benjamin Bhatt, Mohd Halim Mohd Noor, Muhammad Firdaus Akbar, Ahmad Sufril Azlan Mohamed

**Affiliations:** 1 School of Computer Sciences, Universiti Sains Malaysia, Minden, Penang, Malaysia; 2 Department of Information Systems, College of Technological Innovation Abu Dhabi Campus, Zayed University, Abu Dhabi, United Arab Emirates; 3 School of Electrical and Electronic Engineering, Universiti Sains Malaysia, Engineering Campus, Nibong Tebal, Penang, Malaysia; National Institute of Technology Srinagar, INDIA

## Abstract

Path planning is a crucial element of mobile robotics applications, attracting considerable interest from academics. This paper presents a path-planning approach that utilises the Enhanced Firefly Algorithm (EFA), a new meta-heuristic technique. The Enhanced Firefly Algorithm (FA) differs from the ordinary FA by incorporating a linear reduction in the *α* parameter. This modification successfully resolves the constraints of the normal FA. The research involves experiments on three separate maps, using the regular FA and the suggested Enhanced FA in 20 different runs for each map. The evaluation criteria encompass the algorithms’ ability to move from the initial location to the final position without experiencing any collisions. The assessment of path quality relies on elements such as the distance of the path and the algorithms’ ability to converge and discover optimum solutions. The results demonstrate significant improvements made by the Enhanced FA, with a 10.270% increase in the shortest collision-free path for Map 1, a 0.371% increase for Map 2, and a 0.163% increase for Map 3, compared to the regular FA. This work highlights the effectiveness of the Enhanced Firefly Algorithm in optimising path planning for mobile robotics applications, providing potential improvements in navigation efficiency and collision avoidance.

## Introduction

Research on various mobile robot path planning algorithms has been a trending topic. Mobile robots have been used in hazardous environments that are considered harmful to people, such as space exploratory research, radioactive sites, mining sites where explosives and sinkholes can occur, and even deep-sea exploration. Finding a path where the mobile robot can travel safely and efficiently is an essential requirement for any mobile robot system. This highlights the need for research on path-planning algorithms to make the mobile robot move in the shortest collision-free path possible. This prompts the need for this research, as path planning brings many benefits to the area of the mobile robot when the path planned is optimised which include reduced energy consumption, reduced maintenance, and ensuring the safety of the mobile robot.

Ideal path planning will help mobilise the robot effectively and efficiently and ensure the mobile robot’s usability after each use, reducing the cost of repairs and maintenance. However, the majority of the solutions only provide the means to generate a path from the start point to the end point without optimising it. These paths can be optimised in terms of length, smoothness, and even safety of the path generated. However, solutions that are geared up to the area of optimisation can be found at times falling into the local optimal [1–3].

Path planning algorithms have made significant advances in a variety of disciplines in recent years, owing to the growth of autonomous systems and robots. Deep learning-based techniques have gained traction, with convolutional neural networks (CNNs) and recurrent neural networks (RNNs) used to provide end-to-end navigation and map learning for improved path planning. Deep Q-Networks (DQN) and Proximal Policy Optimisation (PPO) are two reinforcement learning approaches that have been used to train agents to navigate complicated and dynamic environments. Multi-agent route planning has grown in importance, notably for coordinating the movements of autonomous vehicles and drones to avoid collisions and optimise trajectories. Researchers are also working on real-time and dynamic situations where path planning algorithms must respond quickly to changes like changing obstructions. To capitalise on the merits of both paradigms, hybrid solutions combining classical search algorithms with machine learning are being investigated. Despite these advances, path planning algorithms continue to encounter common restrictions, such as high-dimensional state spaces, dynamic environments, computational complexity, local optima, uncertainty handling, scalability, and safety and ethics in essential applications [4–7].

In response to the increased demand for efficient and adaptive navigation systems, path planning algorithms have progressed dramatically. Deep learning and reinforcement learning applications have aided advancements in autonomous systems, allowing them to navigate increasingly complicated and dynamic situations. Furthermore, multi-agent path planning has gotten a lot of attention in the context of autonomous vehicles and drones, where collaborative decision-making is required to assure safety and efficiency. The need to reconcile computational complexity with real-time needs remains a key difficulty in real-time adaptation to dynamic situations. Furthermore, tackling difficulties like local optima, sensor data ambiguity, scalability in large-scale systems, and ethical considerations in important applications such as autonomous cars remain a focus of continuing study. As path planning algorithms progress, it is critical to remain current on the newest research and breakthroughs to understand better how these algorithms overcome their inherent limits [8–10].

This study attempts to address the above issue by proposing an enhanced path-planning solution to generate the shortest possible collision-free path to be used for a mobile robot. This enhanced path planning solution will generate a globally optimal path in the area of path length and obtain the solution more efficiently to make it more usable in real-time applications. It is focused on providing an optimal solution to path planning and the usage of meta-heuristic algorithms provides the means to enable that optimal path planning. The aim is to identify the weakness of the meta-heuristic algorithm (in this case, the Firefly Algorithm (FA)), followed by the enhancement of FA to improve its overall performance. The Enhanced Firefly Algorithm (EFA) will then be evaluated on its performance by comparing it with the unaltered/standard FA. To develop an EFA for optimal path planning, the EFA would be required to be able to operate in different map environments to demonstrate its performance and capability.

This research provides a global path-planning solution using an Enhanced Firefly Algorithm (EFA). It focuses on providing an optimal path for mobile robots and helps them minimise their power consumption when travelling the optimal path by introducing a linear decreasing approach on the *α* setting. Besides energy efficiency, the mobile robot will be able to travel safely along the path and minimise the need for unnecessary maintenance. The proposed method will improve its ability to obtain the optimal solution and increase the convergence speed to get the optimal solution, balancing the behaviour of exploration and exploitation. The enhancement used can also be applied to other swarm-type meta-heuristic algorithms, allowing for an improvement in their performance.

## Firefly Algorithm

The Firefly Algorithm (FA) was introduced in 2008, which imitates the mating behaviour of fireflies [11]. Fireflies communicate by varying their light intensity. This light intensity also has an inverse relationship with the distance. The larger the distance, the lower the light intensity. The core concept of the algorithm described by [12] are:

1. There is no gender distinction between fireflies, and attraction is related to the light intensity of each firefly.
2. The attraction between two fireflies is proportional to their brightness and inversely proportional to the distance between fireflies.
3. Brightness of the firefly is determined by the value of the objective function.

**Fig 1:**
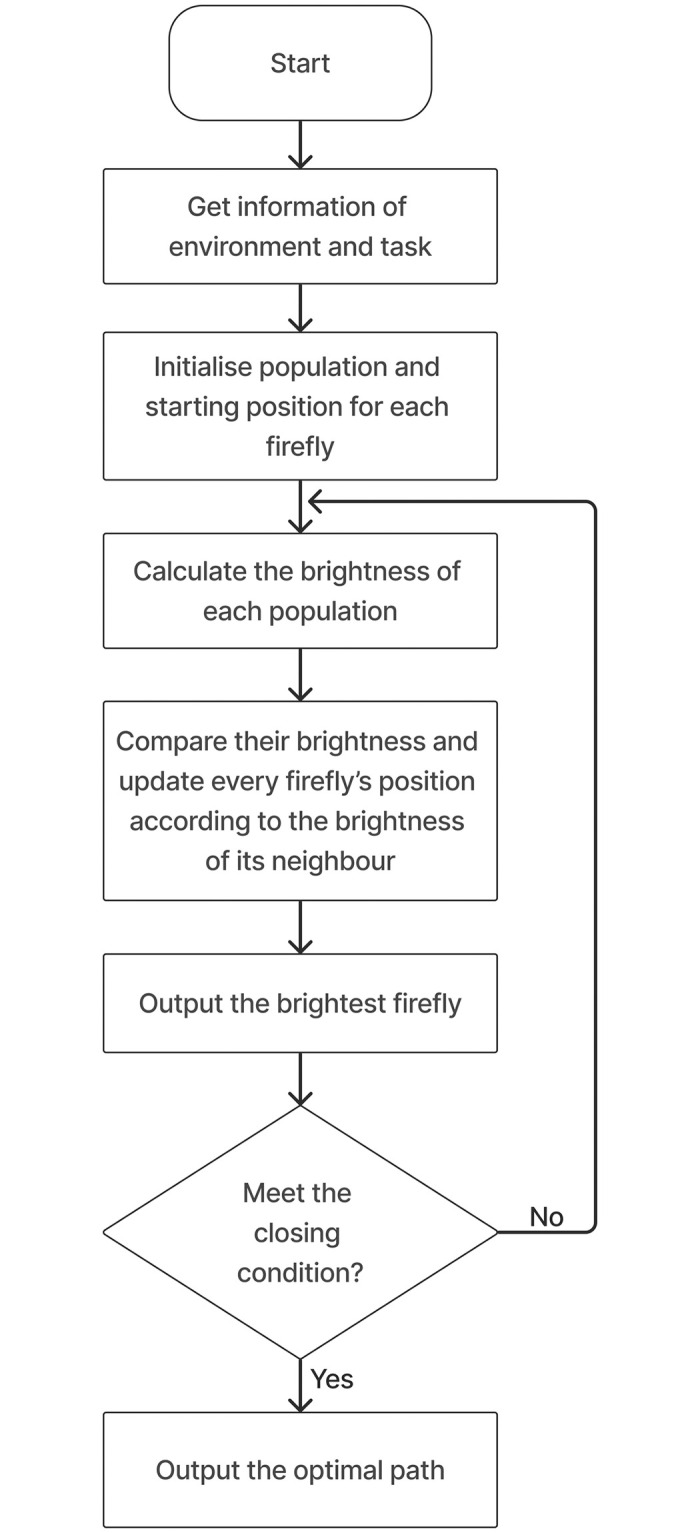
Firefly Algorithm flow chart [24].

The algorithm flow can be referred to in Fig 1. In brief, the algorithm begins by obtaining information about the environment or task. Once completed, the firefly is initialised, and its location across the map is recorded. The transfer function calculates the brightness of each firefly. Once the brightness is set, each firefly will be compared based on its brightness across the neighbouring fireflies. If the target function is reached, the iteration ends. Otherwise, it will continue to calculate the brightness of every firefly and repeat the steps again.

Researchers have been utilising FA in path planning for multiple types of applications [13–16]. [17] utilise FA to achieve multi-objective path planning such as path length, path safety and path smoothness. They demonstrated that by using Firefly alone, it was able to outperform and provide more reliable results compared to the Non-dominated Sorting Genetic Algorithm (NSGA), which is another form of multi-objective path planning but using GA. Another work uses FA to achieve path planning in an unknown environment [18]. It outperformed other approaches such as Neuro-Fuzzy, GA, and Fuzzy-Neural by 6%. [19] proposed adding an additional feature in their algorithm by adding a deletion point when a point is stagnated. This provides better memory control and increased speed to get the optimal solution.

FA has also been used in a hybrid method. For example, [20] introduced a hybrid algorithm that combines FA with particle swarm optimisation (PSO) to enhance convergence speed and solution accuracy, displaying improved performance in complicated optimisation tasks. Furthermore, adaptive techniques have been used to dynamically modify parameters during the optimisation process. [21] proposed an adaptive firefly algorithm (AFA) that modifies attractiveness and randomness parameters depending on iteration, improving the algorithm’s exploration and exploitation capabilities. Furthermore, the use of FA in multi-objective optimisation has made significant progress. Zhang et al. (2019) created a multi-objective firefly algorithm (MOFA) that uses a Pareto-based strategy to successfully manage several competing objectives, leading to considerable improvements in engineering design issues.

The FA’s adaptability has led to its use in a variety of engineering and technology fields. In electrical engineering, FA has been used successfully to optimise the design and operation of electrical systems. [22] used FA to optimise load dispatch in power systems, resulting in cost savings and increased system dependability. In mechanical engineering, [23] used FA to optimise the design parameters of gear mechanisms, leading to improved performance and lower material prices. In civil engineering, FA is used in structural engineering to optimise structure layout and design. [25] optimised steel frame design with FA, resulting in considerable material and construction cost savings.

Due to its durability, the FA is well-suited to tackling complicated data science and machine learning challenges. In feature selection, FA is commonly used to reduce dataset dimensionality while maintaining or enhancing classification accuracy. [26] proposed a feature selection approach based on FA that reduces dataset dimensionality while preserving or enhancing classification accuracy. Furthermore, the technique has been used for data clustering tasks. [27] presented an FA-based clustering algorithm that outperforms existing clustering approaches in terms of accuracy and computing efficiency.

Despite progress, some problems remain in the implementation of FA. The performance of FA can decline as problem complexity increases. Researchers are looking at dimensionality reduction methods and hybrid ways to overcome this issue. Furthermore, FA’s performance is very sensitive to its settings. Therefore, future research will focus on building self-adaptive FA variations that can modify parameters autonomously. FA’s stochastic nature can result in significant computational costs. Hence parallel and distributed computing technologies are being researched to improve its computational efficiency.

To summarise, the Firefly Algorithm has proven considerable promise and flexibility across several fields. Recent upgrades and hybrid techniques have increased its performance and applicability. Continued research into adaptive methods and real-world applications is anticipated to strengthen FA’s status as an effective optimisation tool.

### Limitations of Firefly Algorithm (FA)

Although the Firefly Algorithm (FA) is quite popular as one of the optimisation problem solvers, it still has several limitations. For starters, it frequently demonstrates sluggish convergence, particularly in complicated and high-dimensional problem fields. This is due to the firefly’s intrinsic unpredictability, which might lead to uneven progress during optimisation. Second, FA is prone to premature convergence, finding it difficult to escape local optima in multimodal issues, limiting its potential to identify global solutions. Furthermore, the technique is sensitive to parameter selection, making it difficult to set up successfully for varied domains [28–30].

Another key constraint is the imbalance in behaviour between exploration and exploitation. FA prefers exploitation (refining solutions around the existing best solution) over exploration (looking for new and unexplored portions of the solution space). This imbalance can limit its capacity to properly explore the search space and uncover various answers, which is especially important in complicated, multi-peaked landscapes where exploration is critical. Furthermore, FA lacks a solid theoretical foundation, making it difficult to anticipate its behaviour in different contexts. Furthermore, dealing with limited optimisation issues can be difficult for FA since guaranteeing that created solutions fulfil restrictions might be difficult [28–30].

FA’s scalability is also an issue since its performance may suffer when applied to situations with a high number of variables or constraints. FA may struggle to adapt rapidly and effectively in loud or chaotic contexts when fitness landscapes are continually changing. Furthermore, the computational intensity of FA, particularly when dealing with a large population of fireflies, might be a time and resource-limiting constraint. Finally, FA generally lacks inherent parallelism capabilities, making it unsuitable for parallel and distributed computing settings. To address these limitations, researchers frequently suggest variants and additions to FA, as well as combining them with other optimisation approaches to increase its performance in certain problem areas. To properly handle some of these issues, careful parameter adjustment and problem-specific modifications are required [28].

### Linear decreasing approach

Although path planning approaches have been covered, a gap has been identified to solve the second issue of our research objective, which is solving the problem identified. Although the meta-heuristic algorithm yields better results, sometimes it pre-maturely converges, causing them to fall below the local minimum. The linear decreasing technique has seen success in swarm-type meta-heuristic algorithms in mitigating the shortcoming of these algorithms [31–33]. The linear decrease is a decrement of a value over time. It is useful in key parameters of swarm-type algorithm which focuses on both exploratory and exploitation.

[34] use Krill herd optimisation, using a Linear Decrease in the speed parameter of the Krill algorithm will increase its ability to explore, and as time increases, the speed parameter will decrease over time. This will provide the algorithm with better exploitation. It was tested using an Ackley Function benchmark test and was able to outperform the standard Krill Herd.

Particle Swarm Optimization (PSO) is another swarm-type optimisation algorithm [35]. [36] use an adaptive approach in weighting values to maximise the exploration and exploitation of the optimisation. It was able to perform better by achieving a faster convergence speed and avoiding pre-mature convergence compared to the standard PSO algorithm.

Electromagnetism-like Mechanism (EM) Algorithm used similar concepts such as swarm-like algorithm [37]. Meanwhile, [38] used a modified EM, with gradient-type learning. The algorithm was started by focusing on the exploration of the search space and, with the increase of time, focused on the exploitation, altering the search step size.

### Enhanced Firefly Algorithm (EFA)

Meta-heuristic approaches are chosen over classical approaches due to the efficiency meta-heuristic algorithms have over classical. This can be seen from many literature reviews that classical approaches tend to use more time when the complexity of the problem increases and always achieve local optima [39–41]. Additionally, there are many meta-heuristic algorithms to choose from to achieve our goal. However, for this thesis. The meta-heuristic algorithm of focus will be the Firefly Algorithm (FA). Two versions of the FA were developed for this study, the first being the standard unaltered FA and the next being the enhanced FA with linear decrease. There are three key parameters that affect the FA. The ‘*α*’ parameter affects the movement of the firefly towards a brighter firefly, the ‘*β*’ affects how the firefly perceives the brightness when the distance between the firefly two fireflies is 0, and the ‘*γ*’ which represents the light absorption coefficient.

**Fig 2:**
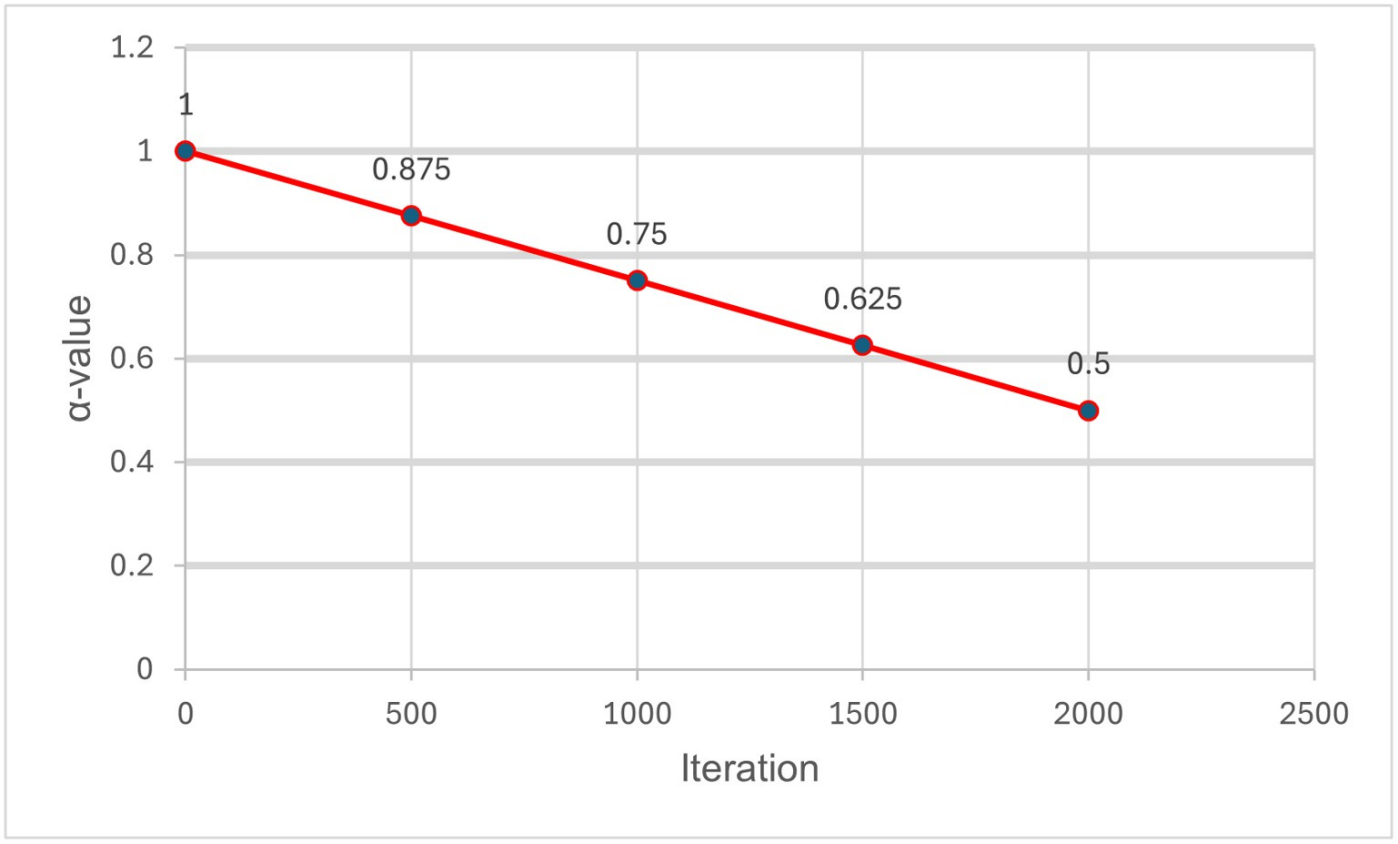
Linear decrease implementation on Enhanced Firefly Algorithm (EFA).

The EFA was modified with a linear decrease in one of the key parameters for this algorithm. The EFA will utilise this technique on the ‘*α*’ parameter of the FA. The ‘*α*’ parameter will start off with a value and will decrease based on the number of iterations. The ‘*α*’ will be set at 1 and will decrease with a step size of 0.125 once the iteration count reaches 500, 1000, and 1500. An example of linear decrement can be seen in Fig 2. This decrement of alpha value per 500 steps was chosen to provide the FA enough time to fully utilise each new alpha value in obtaining the solution.

**Algorithm 1** Firefly Algorithm for Path Planning

1: Initialize firefly population *F* with random positions

2: Set maximum generations *MaxGen*

3: Set absorption coefficient *β*

4: Set attractiveness coefficient *α*

5: **for** *gen* ← 1 to *MaxGen* **do**

6:  **for** each firefly *i* in *F* **do**

7:   **for** each firefly *j* in *F* **do**

8:    **if** *j* is brighter than *i* **then**

9:     Calculate attractiveness *A*_*ij*_ using *α* and distance

10:    Move *i* towards *j* with step size *A*_*ij*_

11:    **end if**

12:   **end for**

13:   Randomly perturb the position of *i*

14:   Evaluate fitness of *i* in path planning context

15:  **end for**

16:  Sort *F* based on fitness (ascending order)

17:  Update brightness of fireflies based on fitness

18:  Calculate *β* based on *gen* and *MaxGen*

19:  **for** each firefly *i* in *F* **do**

20:   **for** each firefly *j* in *F* **do**

21:    **if** *j* is brighter than *i* **then**

22:     Calculate attractiveness *A*_*ij*_ using *α* and distance

23:     Move *i* towards *j* with step size *A*_*ij*_ ⋅ *e*^−*β*⋅distance^

24:    **end if**

25:   **end for**

26:   Randomly perturb the position of *i*

27:   Evaluate fitness of *i* in path planning context

28:  **end for**

29: **end for**

30: Select the best path from the final population *F*

31: **Return** the best path

The following parameter will affect the firefly’s ability to search for space. Starting from a large ‘*α*’ will increase the randomness of the firefly in the search space. This allows the firefly to fully explore the search space, strengthening the swarm-type meta-heuristic on the exploratory. While decreasing the ‘*α*’ will refine the possible solutions, the reduction in step size will emphasise the exploitation side of the swarm-type meta-heuristic. This allows the algorithm to have the best of both explorations of the search space and exploitation of the solutions. The rest of the parameters, such as the ‘*β*’ and the ‘*γ*’, will remain constant throughout the run of the algorithm. The proposed EFA pseudo-code is shown in Algorithm 2. Refer to lines 4 and 5 for linear decrease modification implemented on the algorithm.

**Algorithm 2** Enhanced Firefly Algorithm with Dynamic *α* for Path Planning

1: Initialize firefly population *F* with random positions

2: Set maximum generations *MaxGen*

3: Set absorption coefficient *β*

4: Initialize initial attractiveness coefficient *α*_initial_

5: **for** *gen* ← 1 to *MaxGen* **do**

6:  **for** each firefly *i* in *F* **do**

7:   **for** each firefly *j* in *F* **do**

8:    **if** *j* is brighter than *i* **then**

9:     Calculate attractiveness *A*_*ij*_ using *α* and distance

10:     Move *i* towards *j* with step size *A*_*ij*_

11:    **end if**

12:   **end for**

13:   Randomly perturb the position of *i*

14:   Evaluate fitness of *i* in path planning context

15: **end for**

16:  Sort *F* based on fitness (ascending order)

17:  Update brightness of fireflies based on fitness

18:  Calculate *β* based on *gen* and *MaxGen*

19:  Update *α* dynamically (e.g., *α* = *α*_initial_/(1 + *gen*))

20:  **for** each firefly *i* in *F* **do**

21:   **for** each firefly *j* in *F* **do**

22:    **if** *j* is brighter than *i* **then**

23:     Calculate attractiveness *A*_*ij*_ using *α* and distance

24:     Move *i* towards *j* with step size *A*_*ij*_ ⋅ *e*^−*β*⋅distance^

25:    **end if**

26:   **end for**

27:   Randomly perturb the position of *i*

28:   Evaluate fitness of *i* in path planning context

29:  **end for**

30: **end for**

31: Select the best path from the final population *F*

32: **Return** the best path

The FA will be solving the path planning problem using minimisation. The algorithm will select the shortest path distance generated for each map environment by the objective function ‘Obj’:

$$\begin{array}{c} \text{Obj}=\text{min}(\sum_{i=0}^{n-1}(\sqrt{{\left(x_{i}+x_{i+1}\right)}^{2}+{\left(y_{i}+y_{i+1}\right)}^{2}})) \end{array}$$ (1)

### Experiment setup

This research will be conducted as experimental research encompassing two major stages. The first stage of the experiment was treated as a reference experiment that was conducted using the standard FA. The standard FA utilized fixed values for its ‘*α*’, ‘*β*’, and ‘*γ*’ parameters throughout the experimentation. The result obtained was used as a reference point to evaluate the performance of the algorithm and its effectiveness in the three environmental models. This provided a benchmark for the second stage of the experiment.

The second stage of the experiment was set up as per the first stage of the experiment while using the EFA. The EFA’s ‘*α*’ parameter was linearly decreased while the other two parameters, ‘*β*’ and ‘*γ*’, remained constant throughout the run. The results of this stage of the experiment were used to evaluate its performance from the first stage. Both experiments were conducted by running experiments with 20 sample runs, while a student t-test was used as a statistical analysis method to compare the two samples means. A two-tailed T-test was considered due to the assumption of two samples having equal means, whereas the significance level is at 5% or 0.05 (95% confidence level).

### Map design

There are a few important aspects to the map design, such as the starting point, end point, obstacle position, and obstacle size. This will be represented in an *XY* coordinate view, with the *Y*-axis representing the *X* and *Y* coordinates for the mobile robot, respectively. The blue circles represent obstacles present on the map. The starting point and end point are assumed to be known.

**Fig 3:**
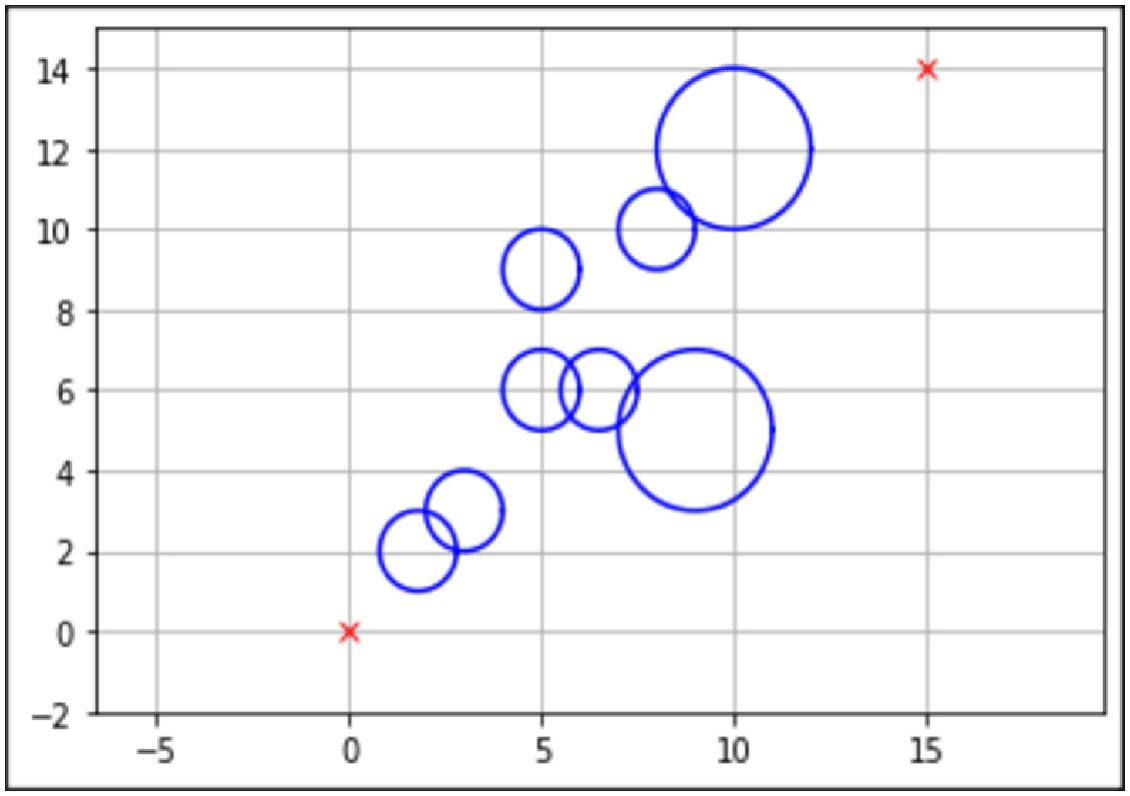
Environment Map 1. The Unit for Length Meter (*m*) with the Starting Point (0,0) and the Endpoint (15,14). This Map Contains Six Static Obstacles with a Radius of 0.5m and Two Static Obstacles with a Radius of 1*m*.

**Fig 4:**
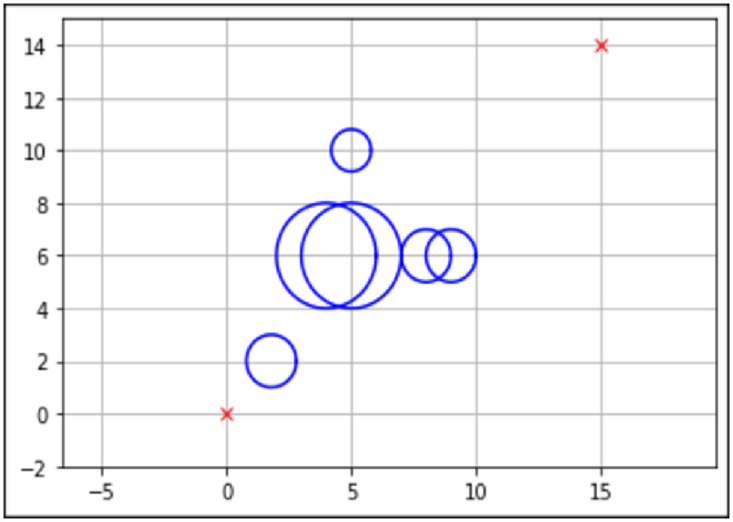
Environment Map 2. The Unit for Length Meter (*m*) with the Starting Point (0,0) and the Endpoint (15,14). This Map Contains One Static Obstacle with a Radius of 0.25*m*, Two Static Obstacles with a Radius of 2*m*, and Three Obstacles with a Radius of 0.5*m*.

**Fig 5:**
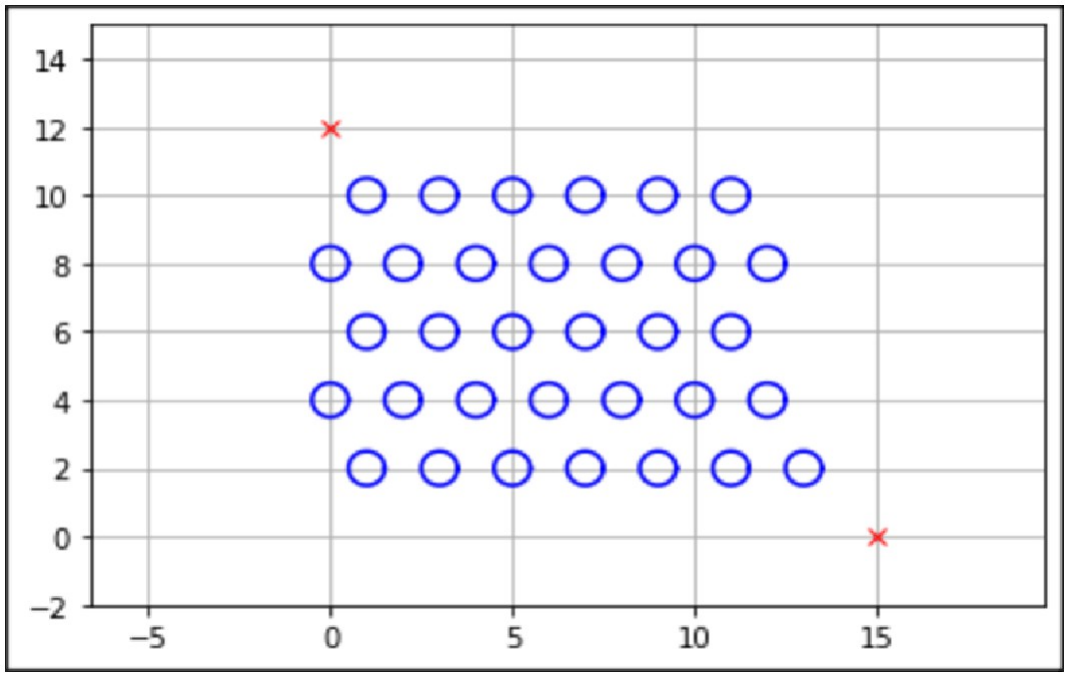
Environment Map 3. Unit for Length Meter (*m*) with the Starting Point (15,0) and the Endpoint (0,12). This Map Contains Thirty-Two Static Obstacles with a Radius of 0.25*m* each.

Three maps have been designed using the information that was gathered from existing literature, as seen in Figs 3–5. The three maps were created to evaluate the performance of the proposed algorithm within different map complexities.

### Experimental parameter values

The FA will use the following parameter values for the experimentation. The iteration, the number of fireflies and alpha values were through experimental procedures can obtain optimal results. This is due to the number of nodal points set for the map allowing for multiple combinations of paths. Thus, using a large population size will maximize the firefly algorithm exploration ability to search the search space for the optimal path. The iteration value of 2000 was used to provide the algorithm with enough iterations to fully utilise each new alpha value.

Each new *α* value will run 500 iterations, sufficient time to obtain an optimal solution as set by [42]. The *β* and *γ* values are obtained from [42] as well. An overview of the summary can be referred to in Table 1.

**Table 1:** Parameter settings for standard FA and enhanced FA [42].

Parameter	Standard FA	Enhanced FA
Iteration/Generation	2000	
Number of fireflies	20	
*α* initial	0.5	1
*α* final	0.5	0.625
*β*	1	
*γ*	0.5	

### Path planning simulation experiment setup

This path planning experiment was run on Lenovo Ideapad Y580 equipped with Intel Core i7-3610QM 72, NVIDIA GeForce GTX 660M (2GB GDDR5) 128, and 4GB DDR3, 1333 MHz. The performance measure will include the following parameters such as total path distance, algorithm run time, and algorithm convergence. The optimisation algorithm underwent benchmark testing to evaluate the performance.

#### Total path distance

This was calculated using the distance between each consecutive distance of the final path generated in each generation. The objective is to obtain the shortest path possible during each run for the respective maps. This used the equation shown in Eq (2).

$$\begin{array}{c} D=\sum_{i=0}^{n-1}(\sqrt{{\left(x_{i}+x_{i+1}\right)}^{2}+{\left(y_{i}+y_{i+1}\right)}^{2}}) \end{array}$$ (2)

#### Algorithm convergence

The iteration where the best path is obtained was recorded. The objective of this performance metric is to obtain the optimal solution with the fewest iterations for the Firefly algorithm. The recorded value was averaged over the 20 independent runs to get the convergence speed of both the standard and enhanced Firefly Algorithms.

#### Optimisation function benchmark testing

In order to evaluate the effectiveness of the optimization performed on the enhanced and standard functions, a benchmark test was conducted using the Ackley Function and Michalewicz Function [43, 44].

## Results and discussion

### Total path distance

This is the primary performance measure for both the standard FA and enhanced FA, as it is the algorithm’s objective function. The aim was to get the shortest path from the start point to the endpoint from the three map environments presented in the previous section.

The best path generated for both the standard and enhanced FA can be seen by the red line in Figs 6 and 7 for Map 1, Figs 8 and 9 for Map 2 and Figs 10 and 11 for map 3. The results summary can be seen in Table 2.

**Fig 6:**
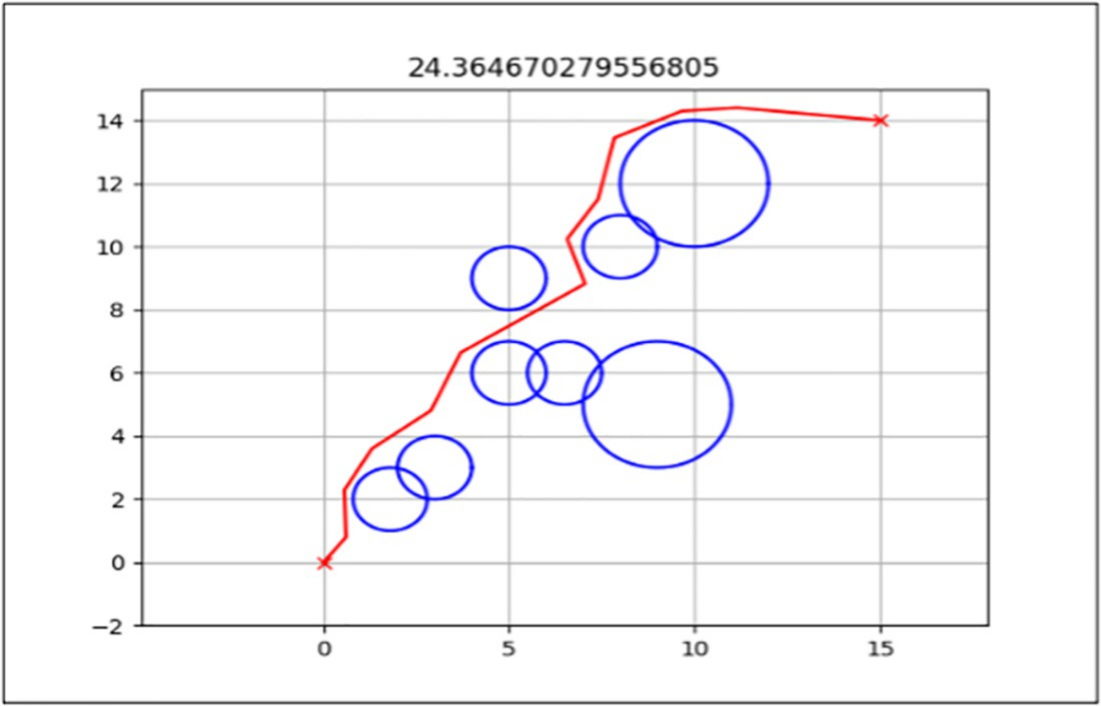
The path generated by the standard Firefly Algorithm (FA) between the starting point (0,0) and the endpoint (15,14) for Map 1 with a total distance of 24.364*m*. This map contains six static circles with a radius of 0.5*m* and two static circles with a radius of 1*m*.

**Fig 7:**
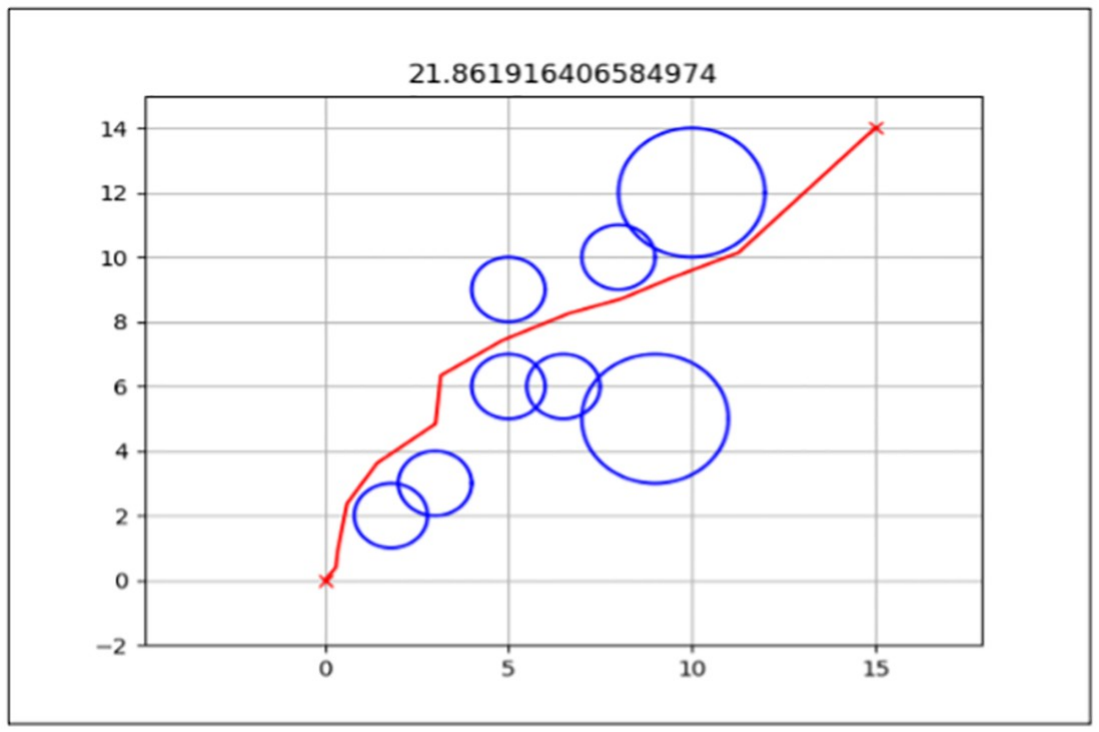
The path generated by the Enhanced Firefly Algorithm (EFA) between the starting point (0,0) and the endpoint (15,14) for Map 1 with a total distance of 21.862*m*. This map contains six static circles with a radius of 0.5*m* and two static circles with a radius of 1*m*.

**Fig 8:**
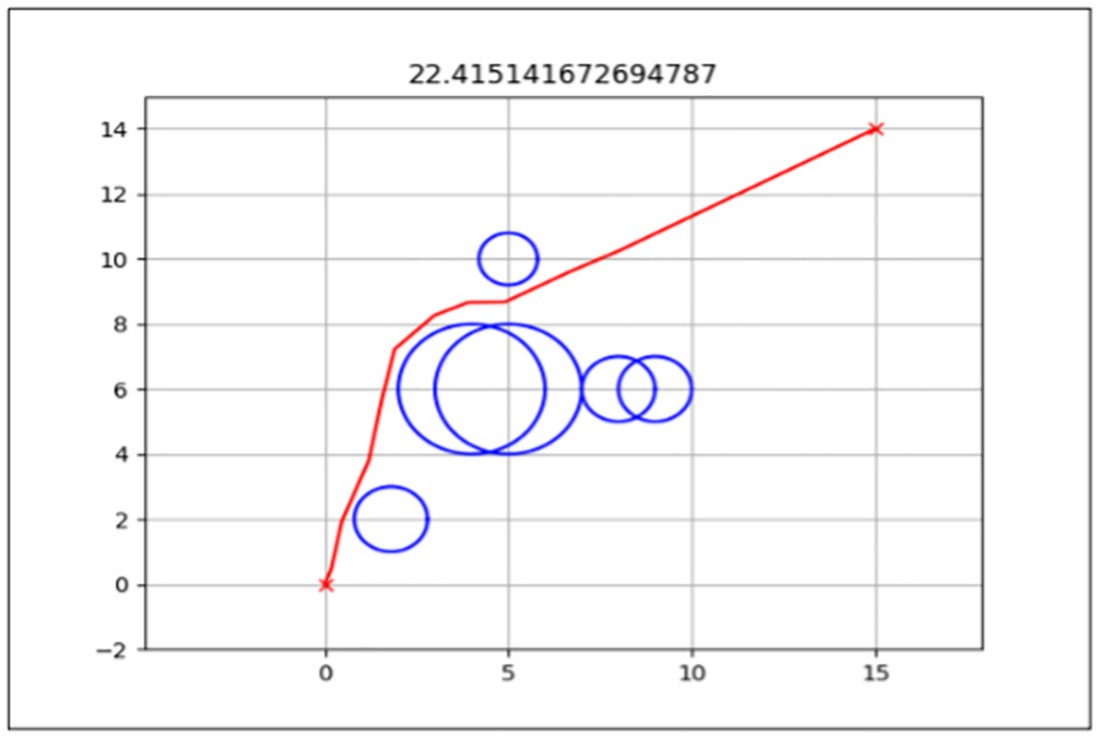
The path generated by the standard Firefly Algorithm (FA) from a start point of (0,0) and endpoint (15,14) for Map 2 with a total distance of 22.415*m*. This map contains six static obstacles with a radius of 0.5*m* and two static obstacles with a radius of 1*m*.

**Fig 9:**
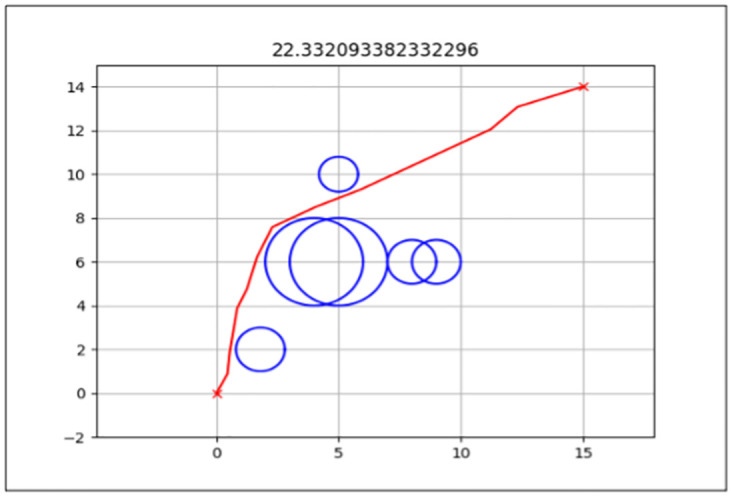
The path generated by the Enhanced Firefly Algorithm (EFA) from a start point of (0,0) and endpoint (15,14) for Map 2 with a total distance of 22.332*m*. This map contains six static obstacles with a radius of 0.5*m* and two static obstacles with a radius of 1*m*.

**Fig 10:**
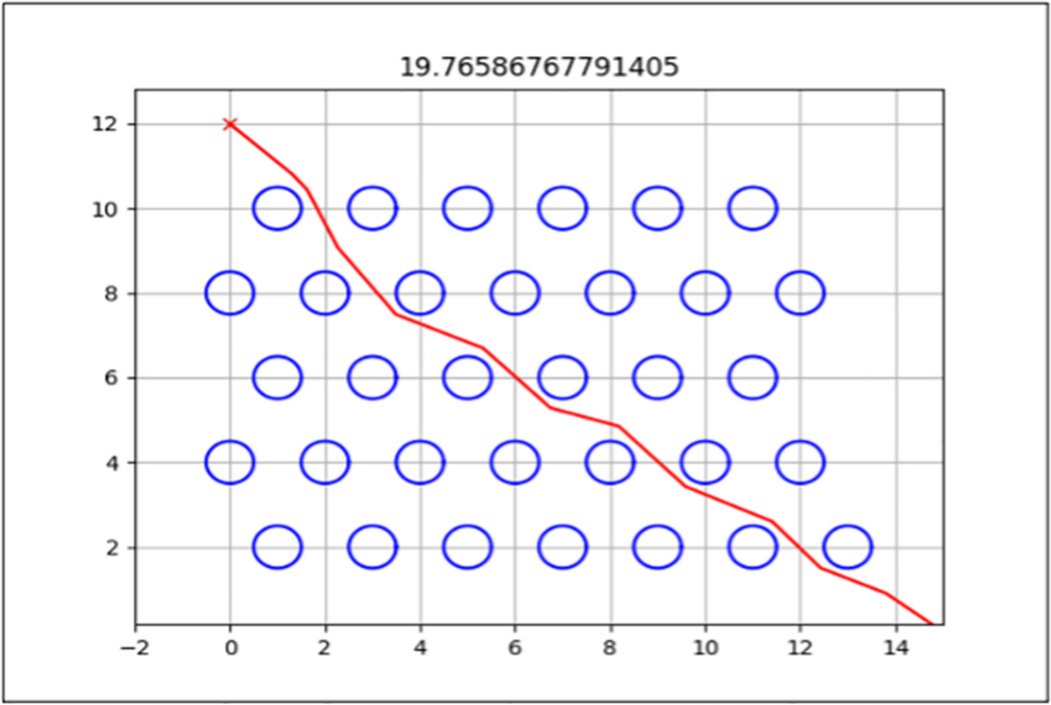
The path generated by the Standard Firefly Algorithm (EFA) from a start point (15,0) with an endpoint of (0,12) for Map 3 with a total distance of 19.765*m*. This map contains 32 static obstacles, each with a radius of 0.25*m*.

**Fig 11:**
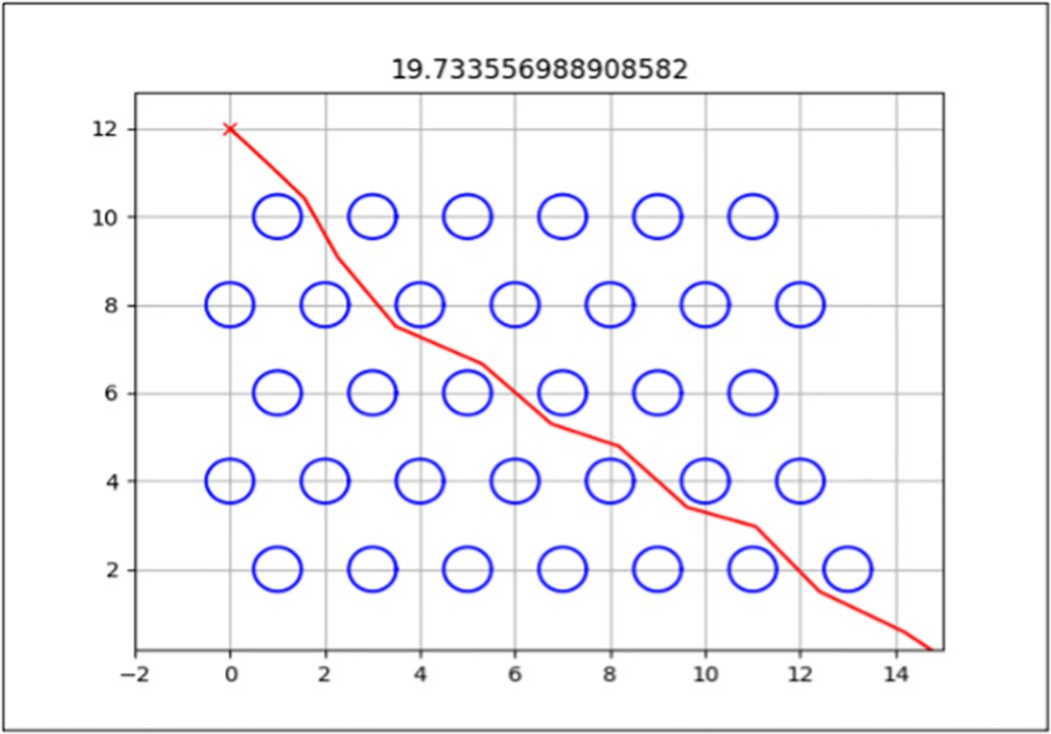
The path generated by the Enhanced Firefly Algorithm (EFA) for Map 3 is from a start point (15,0) to an endpoint of (0,12), with a total distance of 19.734*m*. This map contains 32 static obstacles, each with a radius of 0.25*m*.

**Table 2:** Result of the standard FA against enhanced FA on the travelled distance.

Map	Measurement (*m*)	Standard FA	Enhanced FA
Map 1	Best (Shortest)	24.364	21.862
	Average	26.061	24.637
	Worst	32.178	25.773
	Standard Deviation	±1.916	±0.817
Map 2	Best (Shortest)	22.415	22.332
	Average	22.634	22.515
	Worst	23.111	22.784
	Standard Deviation	±0.169	±0.113
Map 3	Best (Shortest)	19.765	19.734
	Average	19.890	19.837
	Worst	20.186	19.983
	Standard Deviation	±0.133	±0.0804

The results in Table 2 show the best path obtained by the EFA is reduced by 10.270%, 0.371%, and 0.163% for Map 1, Map 2, and Map 3, respectively, as compared to using the standard FA. Additionally, the EFA reduced the average path by 5.463%, 0.528%, and 0.265% for Map 1, Map 2, and Map 3, respectively. It is observed that an improved performance of the Firefly algorithm in this minimisation problem was due to the linear decrease.

It allowed the algorithm to focus on exploring the map for any possible paths, and once the algorithm reaches close to 2000 iterations, the alpha value reduces, allowing the algorithm to focus on refining the solutions. The refinement of the solution is done by minimising the step size of the firefly, causing the solution to gain more precision. This can also be observed with the lower standard deviation of the enhanced FA on all three maps. The solutions generated are more consistent with previous runs demonstrating the refinement of the solutions when using the enhanced FA.

The study compares the performance of the Enhanced Firefly Algorithm (FA) to the conventional FA over several maps, determining their efficiency in terms of error reduction. Analysing the findings, the Enhanced FA regularly outperforms the normal FA in error reduction, as seen by reduced standard deviation values across all maps. The % error reduction, obtained by comparing the standard deviation of the Enhanced FA to that of the conventional FA, shows considerable improvements. For example, on Map 1, the Enhanced FA achieves an amazing error reduction of roughly 57.31% when compared to the Standard FA. Similarly, on Maps 2 and 3, the Enhanced FA reduces percentage errors by approximately 33.73% and 39.62%, respectively. These studies demonstrate the effectiveness of the Enhanced FA in reducing mistakes and improving optimisation performance. Overall, the study shows an average error efficiency of roughly 43.22%, indicating the significant advances made by the Enhanced FA in tackling optimisation issues and enhancing solution quality.

### Convergence rate

The iteration count for the shortest path was recorded for each run. The aim of this performance test was to obtain the solution with the least iteration count. As seen in Table 3, the average iteration in obtaining the shortest path or arriving at an optimal solution varied across the standard FA and Enhanced FA. It was observed that the standard FA had a -8.659% iteration decrease in obtaining the optimal results on Map 1 compared to the enhanced FA. This shows that the standard FA could find the optimal solution quicker than the enhanced FA. Whereas the enhanced FA had iteration improvements of 27.97% and 5.94% for Map 2 and Map 3, respectively, demonstrating the enhanced FA was able to get the optimal solution with fewer iterations compared to the standard FA.

**Table 3:** Summary of average iterations optimal solution found.

Map	Standard FA	Enhanced FA
Map 1	602.02	654.3
Map 2	1063	766
Map 3	1134	1066

### Benchmark testing between standard FA and enhanced FA

Benchmark functions are useful to evaluate new algorithms’ general performance. Two standard benchmark functions were used to evaluate the performance of the proposed enhanced FA and the standard FA. The algorithm uses the parameter mentioned in Table 1.

As seen in Table 4, the EFA can achieve the same best global minimum result compared to the standard FA for both Ackley and Michalewicz functions. On the Ackley function, the enhanced algorithm could converge into the global minimum in all 20 runs with an average convergence of 79 iterations. Meanwhile, with the Michalewicz functions, the standard FA performed better in terms of obtaining a better resolution on the average fitness value than the enhanced FA. However, the enhanced FA achieved a better convergence rate to obtain the global optimum.

**Table 4:** Ackley and Michalewiz benchmark function results.

Function	Statistics	Standard FA		Enhanced FA	
		Fitness	Iteration	Fitness	Iteration
Ackley	Best	0	1	0	1
	Average	2.218	25.500	0	17.200
	Worst	5.401	99	0	79
	Standard Deviation	±1.986	±36.620	±0	±24.260
Michalewicz	Best	-1.602	25	-1.602	8
	Average	-1.602	53.500	-1.602	36.800
	Worst	-1.602	96	-1.602	69
	Standard Deviation	±0.001	±20.650	±0	±22.840

The reduced iterations in achieving the solution demonstrate a faster convergence rate in obtaining the global minimum. The linear decrease in alpha value contributes to fast convergence. Furthermore, the improvement of the algorithm in obtaining the global minimum for all 20 runs demonstrates the high alpha value allows for the maximisation of the exploration. This prevents premature convergence and falls into the local optima.

## Analysis

As observed from the results, the EFA can provide a better solution than the standard FA in general. An improvement of 5.463%, 0.528%, and 0.265% for Map 1, Map 2, and Map 3 are found for the EFA compared to the standard FA. EFA also obtained a shorter average distance for 20 runs. As there was an improvement in the path obtained, a t-test was used as a statistical analysis method to assess the statistical significance by comparing the two-sample means. A two-tailed t-test is considered due to the assumption of two samples having equal meaning, whereas the significance level was set at 5% or 0.05 (95% confidence level).

As seen in Table 5, a statistical significance can be seen when using the algorithms on Map 1 and Map 2, as both the P values were less than 0.05. However, Map 3 does not demonstrate this significance. This could be due to the performance of the standard, and the enhanced FA achieved similar results in terms of the best path with only a difference of 0.163% on a best-to-best path comparison.

**Table 5:** P-value results for the two-tailed t-test.

Map	P(T≤ t) two-tail
Map 1	0.00666
Map 2	0.01290
Map 3	0.15900

Based on the results obtained from the experiments, the map environment plays a big role in the performance of both algorithms. A more direct obstacle that interferes with a possible straight line from the start to the end point tends to have a more significant impact, as seen in Maps 1 and 2. Meanwhile, in Map 3, the obstacles are more spread out and do not interfere with the direct start-to-end goal line, tending to achieve similar results for both standard and proposed enhanced FA. The benchmark testing provides a good guideline on where the algorithm performs for both Ackley and Michalewicz functions.

The proposed Enhanced FA improvement on the alpha ‘*α*’ parameter using a time-variant technique allows for a larger search space for each firefly. This method will provide a more diverse solution for the FA, as with a large ‘*α*’, there is an increase in the randomness of the step size. As the iteration count progresses, the decrements of the ‘*α*’ present an opportunity to refine the solution into smaller step sizes.

**Fig 12:**
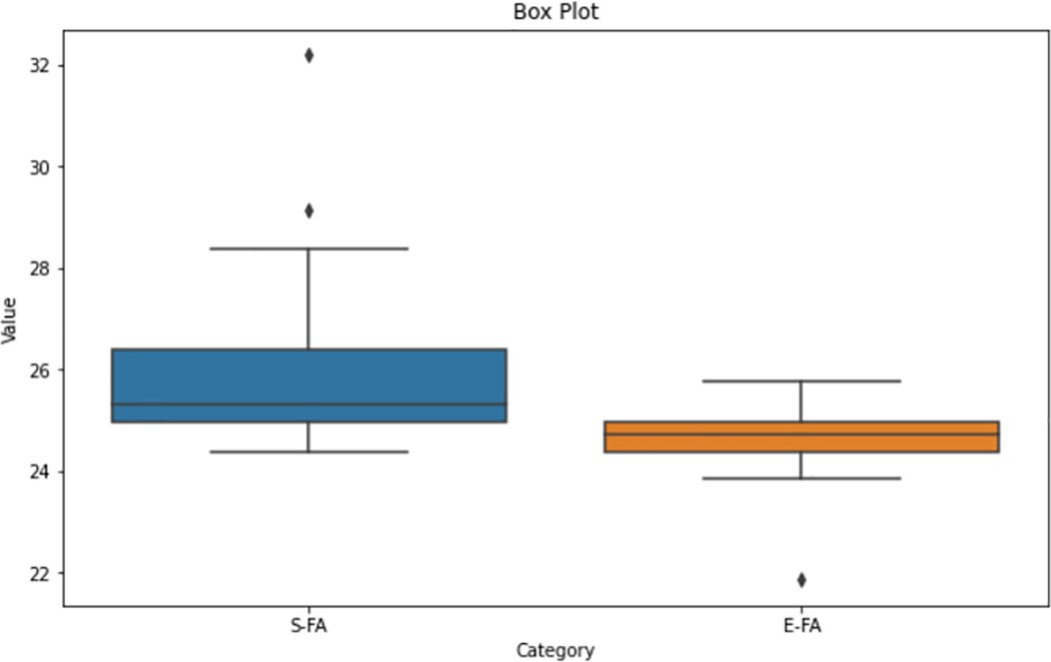
Box plot of length between standard FA and enhanced FA for Map 1.

**Fig 13:**
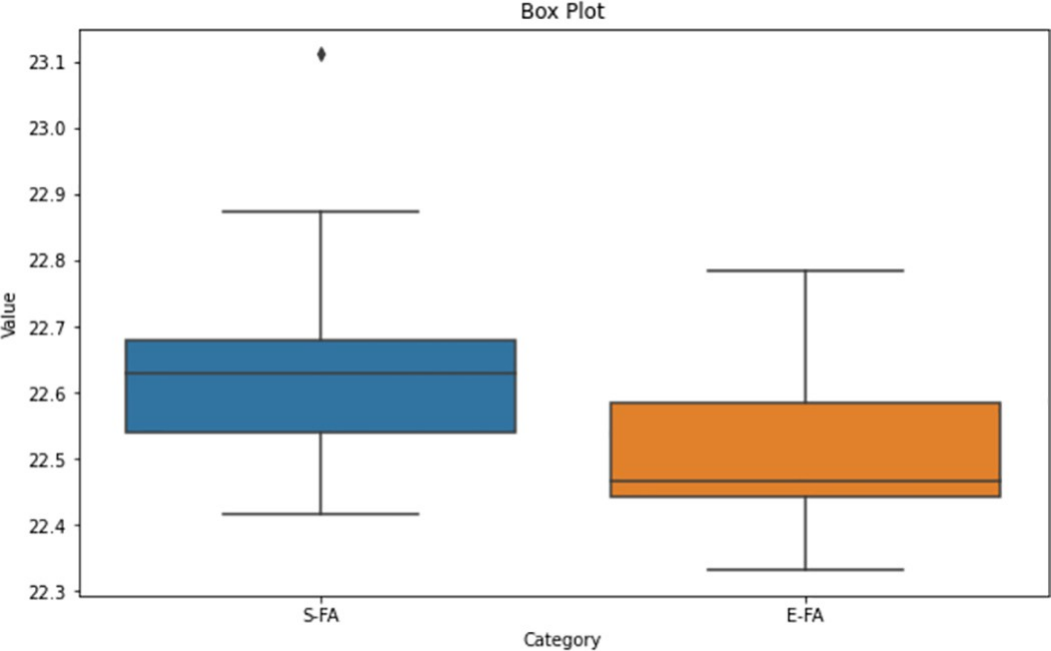
Box plot of length between standard FA and enhanced FA for Map 2.

**Fig 14:**
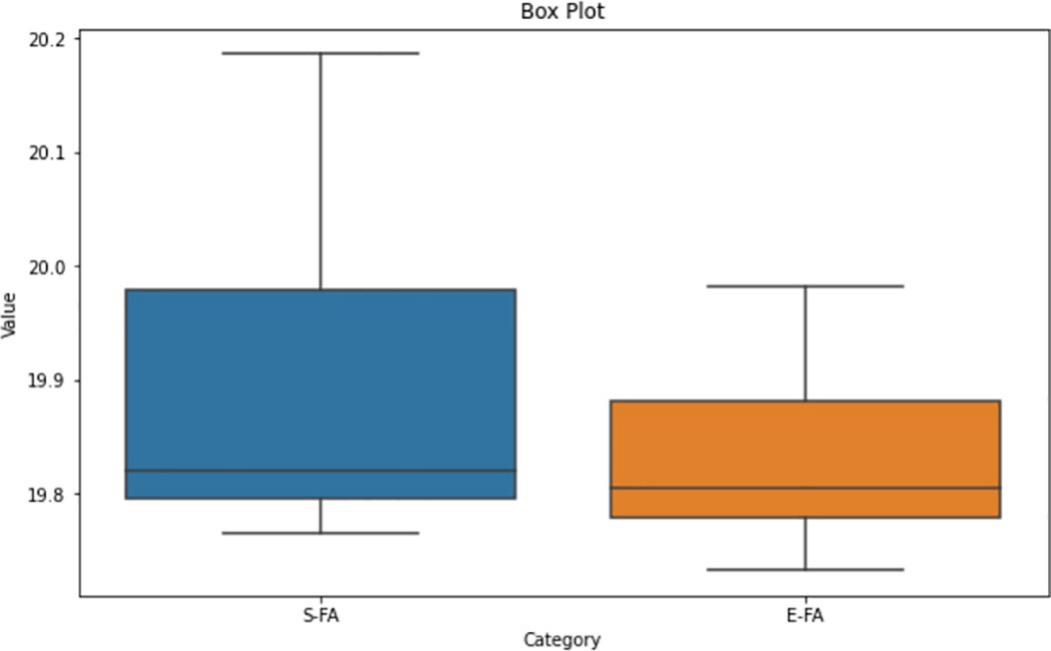
Box plot of Length between standard FA and enhanced FA for Map 3.

Upon analysing the box plot displayed in Figs 12–14, it is apparent that both the Standard FA and Enhanced FA columns exhibit a substantially symmetrical data distribution. This is indicated by the median line positioned in each box’s centre. The inter-quartile ranges for both columns appear to be of equal lengths, indicating that the data has comparable variability. No apparent anomalies exist in any of the columns since all data points are within the range indicated by the box plots. The median values for the Standard FA and Enhanced FA columns are quite close, suggesting comparable core trends.

**Fig 15:**
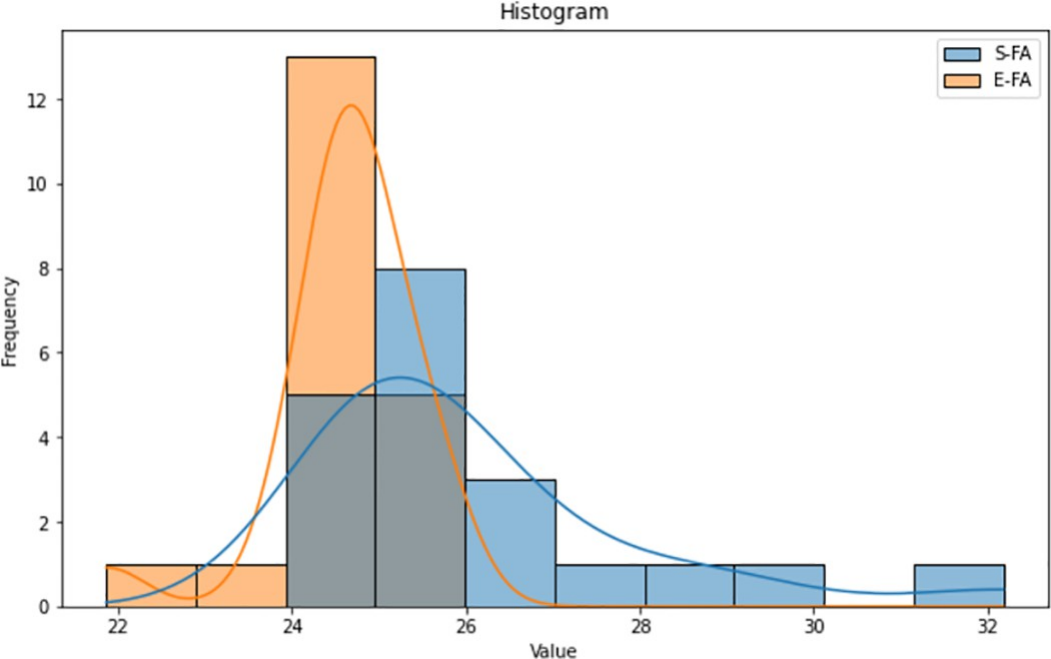
Histogram plot of length between standard FA and enhanced FA for Map 1.

**Fig 16:**
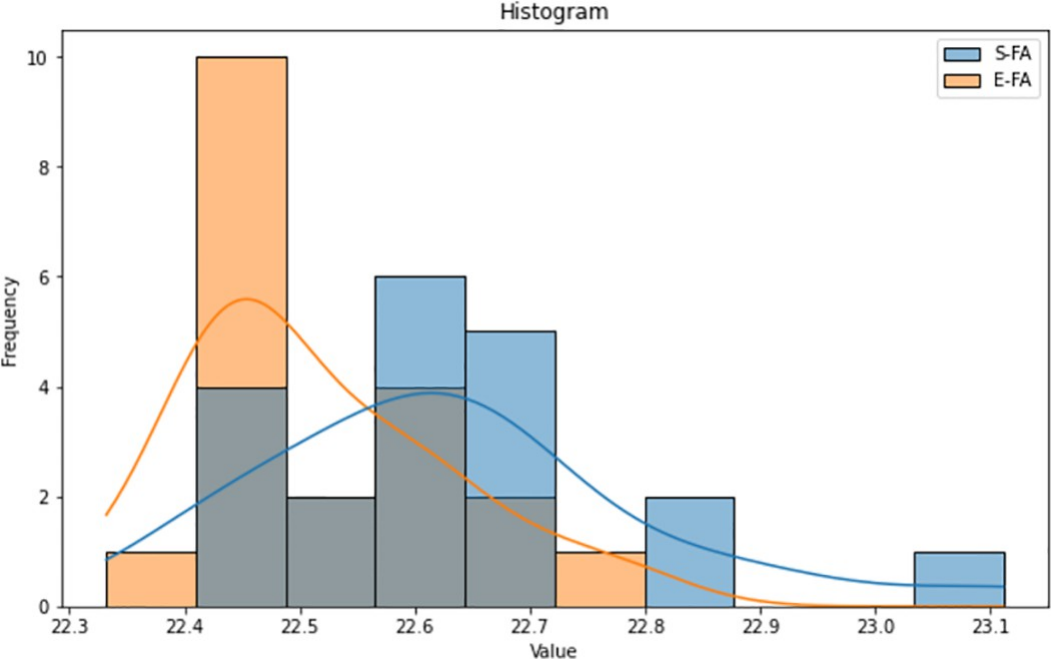
Histogram plot of length between standard FA and enhanced FA for Map 2.

**Fig 17:**
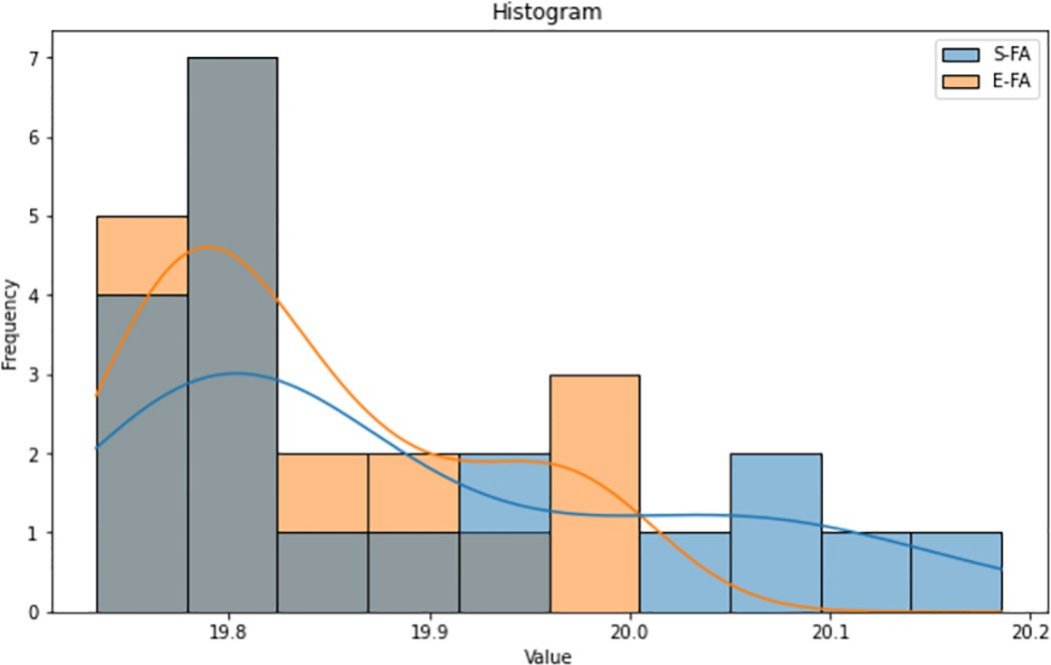
Histogram plot of length between standard FA and enhanced FA for Map 3.

The histograms (Figs 15–17) for both the Standard FA and Enhanced FA columns exhibit almost symmetrical distributions, with a prominent peak in the data range’s centre. Both histograms exhibit unimodal distributions, suggesting that the data in each column have a solitary mode or peak. The histograms for both columns have similar forms, indicating comparable underlying distributions. The values for both columns have a rather limited range, with most values contained inside a tight interval.

According to the observation of the box plot and histogram in Figs 12–17, it appears that the distributions of the S and E columns are relatively comparable. These visualisations do not exhibit any discernible disparities in their distributions. However, additional statistical analysis, such as mean, variance, and hypothesis testing calculations, may be required to establish the parallels or differences between the two columns with greater rigour.

## Conclusion

Path planning has been gaining traction with increased technological advances, such as the wide usage of drones, mobile robots, and autonomous vehicles. To prolong the lifespan of the mechanical aspects of these devices, effective path planning is necessary to minimize error and redundant movements. Current developments in path planning for mobile robots are at an all-time high, involving classical and meta-heuristic approaches. However, the meta-heuristic approach has been gaining a fair amount of attention due to its optimisation capabilities, less intensive computational requirement, and flexibility of the algorithms. This study utilises the Firefly Algorithm to solve the path planning problem. The FA was inspired by observing fireflies mating.

Through observation, the FA can be defined with three rules which are there is no gender distinction between fireflies, attraction is related to the light intensity of each firefly, the attraction between two fireflies is proportional to their brightness and inversely proportional to the distance between fireflies and brightness of the firefly is determined by the value of the objective function. The FA uses the basis of fireflies to move around the search space randomly, and the objective function defines the firefly brightness. This makes it suitable for path planning as the random movement of the firefly can diversify the solutions. Three parameters control each firefly’s movement: alpha, ‘*α*’, beta, ‘*β*’, and gamma, ‘*γ*’.

The Enhanced FA (EFA) utilised a time-variant constant technique on the alpha, ‘*α*’ parameter. This provides a larger search space for the firefly to search for a solution as the step size depends on the alpha, ‘*α*’ parameter. By starting with a large alpha, ‘*α*’ value, more solutions can be found. As the algorithm continues the iterations, the alpha, ‘*α*’ value decreases.

This provides a more refined result of the possible path found with the large alpha values. Aside from getting a better solution, it will quicken the convergence of obtaining the optimal solution. After completing the experiments of this study, the proposed enhanced FA was found to be more successful in obtaining a better solution than the standard FA. The reduction in the path found was 10.27%, 0.371%, and 0.163% for Map 1, Map 2, and Map 3, respectively.

Additionally, a reduction of iterations to converge into getting the optimal solution was observed for the EFA. The distance results were justified with statistically significant results compared to the standard FA. The proposed enhanced FA was observed to be a more effective and robust solution upon testing with the simulated map environments.

Based on this study, the possible future works to be done are as follows:

(a) The Enhanced Firefly Algorithm can be improved to include multi-objective path planning. This will include other criteria that may be important to path planning, such as the safe distance of the mobile robot from obstacles and the smoothness of the path generated. Additionally, consideration of the environment in 3D, such as terrain, is needed to create more efficient path planning. This study only focused on travelled distance as the objective and was conducted in 2D-dimensional space.
(b) This study uses static obstacles when planning for the mapping environment. Future work could consider including dynamic or moving obstacles, allowing path planning to consider real-world applications and consequently increasing the complexity of the problem.
(c) The current approach currently uses a global path planning method where information is provided to the algorithm beforehand. Certain modifications can make the approach more robust and flexible in local path planning where information on the map is not provided.
